# Synthesis, Hydrolytic Stability and In Vivo Biological Study of Bioconjugates of the Tetrapeptides FELL Containing Pyrrole Moiety

**DOI:** 10.3390/biomedicines11123265

**Published:** 2023-12-09

**Authors:** Boryana Borisova, Stanislava Vladimirova, Hristina Nocheva, Marie Laronze-Cochard, Stéphane Gérard, Stoyko Petrin, Dancho Danalev

**Affiliations:** 1 Biotechnology Department, University of Chemical Technology and Metallurgy, 8 Kliment Ohridski Blvd, 1756 Sofia, Bulgaria; boriana.borisowa@gmail.com (B.B.); stpetrin@uctm.edu (S.P.); 2 Organic Synthesis Department, University of Chemical Technology and Metallurgy, 8 Kliment Ohridski Blvd, 1756 Sofia, Bulgaria; vladimirova.s@uctm.edu; 3 Department of Physiology and Pathophysiology, Faculty of Medicine, Medical University-Sofia, Sv. Georgi Sofiyski Blvd 1, 1431 Sofia, Bulgaria; hndimitrova@medfac.mu-sofia.bg; 4 Institut de Chimie Moléculaire de Reims (ICMR)—UMR CNRS 7312, Université de Reims Champagne-Ardenne, UFR Pharmacie, 51 rue Cognacq-Jay, 51100 Reims, France; marie.cochard@univ-reims.fr (M.L.-C.); stephane.gerard@univ-reims.fr (S.G.)

**Keywords:** bioconguates, FELL, pyrrole, peptides, analgesic activity, hydrolytic stability

## Abstract

Background: Bioconjugates are promising alternatives for the multiple targeting of any disease. Pyrrole heterocycle is well known with many activities and is a building block of a lot of medical drugs. On the other hand, peptides are short molecules with many advantages such as small size, ability to penetrate the cell membrane and bond-specific receptors, vectorizing potential, etc. Thus, hybrid molecules between peptide and pyrrole moiety could be a promising alternative as an anti-pain tool. Methods: New bioconjugates with a general formula Pyrrole (α-/β-acid)-FELL-OH (NH_2_) were synthesized using Fmoc/OtBu peptide synthesis on solid support. HPLC was used to monitor the purity of newly synthesized bioconjugates. Their structures were proven by electrospray ionization mass spectrometry. The Paw Pressure test (Randall–Selitto test) was used to examinate the analgesic activity. Hydrolytic stability of targeted structures was monitored in three model systems with pH 2.0, 7.4 and 9.0, including specific enzymes by means of the HPLC-UV method. Results: The obtained results reveal that all newly synthesized bioconjugates have analgesic activity according to the used test but free pyrrole acids have the best analgesic activity. Conclusions: Although free pyrrole acids showed the best analgesic activity, they are the most unstable for hydrolysis. Combination with peptide structure leads to the hydrolytic stabilization of the bioconjugates, albeit with slightly reduced activity.

## 1. Introduction

Therapeutic bioconjugates are described as a breakthrough in a variety of different drug development fields for their specific pharmaceutical properties [1,2,3]. Often, they bring forth the positive features of both parts of the molecule. In addition, they allow for the modulation of simultaneously different targets related to a disease. Thus, the main aim of creating bioconjugates is the search of a synergic effect between both parts of the molecule. Such kinds of molecules can be used in a highly targeted therapy for selective delivering of therapeutic agents to the specific cells or tissues, i.e., vectorization of the main effect, minimizing damage to healthy cells and reducing side effects. Briefly, the bioconjugates are a combination of two or more different chemical entities linked by covalent bond. It is expected that, after introducing the thus obtained molecule into the body, thanks to some structural features, it will reach the necessary place to perform a programed action after activation by a suitable enzyme run or binding to a specific receptor due to the well-known vectorizing potential of the peptides [4,5,6]. If at least one of the building entities in the molecule is a biologically active component (small heterocyclic molecule, proteins, peptides, etc.) the term “bioconjugates” can be used for the newly formed compound. The peptide–drug conjugates possess larger therapeutic potency and application than antibody–drug conjugates [7,8]. Their lower molecular weight and short amino acid sequences could lead to several improvements such as more flexible structure, higher drug loading and enhanced cellular penetration. Furthermore, the application of bioconjugates is gaining popularity currently due to the creation of an opportunity to treat different infections and diseases [3,9]. Herein, the described strategy for bioconjugate creation was used for the development of a new dual entity, combining a heterocyclic scaffold to a peptide sequence, for pain treatment.

Pain is often considered a global burden that affects people around the world, regardless of their age, gender, race, etc. [10]. Chronic pain often leads to different states such as depression, inability to work, disrupted social relationships, and even suicidal attempts. Moreover, in 2022, the European Pain Federation mentioned a statistic where 1 in 5 European adults report moderate or severe chronic pain, which places pain at the forefront of personality disorders [11]. On the other hand, pain is defined as a symptom which accompanies a wide range of pathologies like musculoskeletal, neurological, gastrointestinal [12], as well as some infectious diseases such as HIV, SARS-CoV-2 and herpes [13]. Pain is recognized as a complex problem due to the multiapproach of analgesia. It accompanies different medical conditions. Its effective management often requires a comprehensive way involving a combination of several medications [14]. The selection of an analgesic treatment depends on the type and intensity of pain, the underlying cause, and individual patient factors but this is still not complex enough. Many different molecules are currently used in a medicinal practice for pain treatment like opioids, non-steroidal anti-inflammatory drugs (NSAIDs), corticosteroids, cyclooxygenase (COX) inhibitors, etc. [14,15,16]. Unfortunately, these medical drugs are often defiled by some undesired effects, such as nausea, respiratory depression, constipation, significant adverse effects on the gastrointestinal tract, etc. [17,18]. Thus, society is in a great need of new analgesics to limit the consequences of this public health issue and to overcome the undesired side effects of those medicaments currently used in the practice. Briefly, regardless of the source, pain is an uncomfortable feeling that often leads to discomfort and functional limitations [19]. Thus, conjugation of some known chemical compounds with a well-proven analgesic effect into hybrid structures with active peptide moiety seems to be an alternative for the creation of new molecules with better directionality and synergic effect as promising opioid agents.

**Figure 1:**
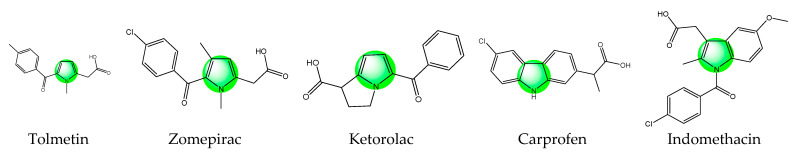
Chemical structures of some pyrrole containing medicinal drugs with analgesic activity.

The heterocycles are among the most used classes of organic compounds in medicinal chemistry [20]. Due to their specific features the molecules of this class have gained immense attention as ingredients of cosmetics, polymers, solvents, antioxidants, etc. [21]. In addition, many heterocyclic molecules are currently in use for the treatment of different diseases [22,23,24]. Pyrrole is a main representative of the thus mentioned heterocyclic compounds. Well-known drugs possessing such a pyrrole scaffold with analgesic activity are presented in Figure 1.

Tolmetin sodium, a pyrrole acetic acid derivative, is a nonsteroidal anti-inflammatory and analgesic drug recommended in the management of rheumatoid arthritis [25]. Zomepirac sodium is an orally effective nonopioid analgesic that can relieve mild to severe pain. It is more effective than aspirin or codeine alone and is as effective as analgesic combinations containing codeine or other narcotics [26]. Ketorolac is one more non-steroidal anti-inflammatory drug that belongs to this class. This drug is available as an oral tablet, injection, nasal spray, and eye solution. Due to its analgesic properties, this drug is used for the treatment of rheumatoid arthritis, postoperative pain, osteoarthritis, menstrual disorders, and for spondylitis. Carprofen is a pyrrole analog and non-steroidal anti-inflammatory drug used for arthritic symptoms. Previously, carprofen was used for the treatment of gastrointestinal pain and nausea. Later, it was banned due to its toxicity [27]. Indomethacin is also a nonsteroidal anti-inflammatory drug with potent antipyretic, analgesic, and anti-inflammatory activity that has been effectively used in the management of mild-to-moderate pain since the mid-1960s. It is commonly prescribed for the relief of acute gouty arthritis pain, but has demonstrated efficacy in the treatment of various other painful conditions [28]. All these medicaments prove that the addition of an active heterocyclic moiety could be advantageous in the design of new hybrid molecules with targeted activity. The pyrrole is often described as a “resourceful small molecule” which possesses many activities among which are analgesic and anti-inflammatory activities [29,30]. This makes the drug-like pyrrole a possible alternative to be combined with peptides with similar activity in search of a synergistic effect between both parts of the hybrid entity in order to create new multitarget bioconjugates.

In this context, tetrapeptide sequence H-FELL-OH is known from the literature as an anti-inflammatory agent. The study of Laurent et al. revealed that it reduces by 51% the total number of white blood cell accumulation in the bronchoalveolar fluid in lungs [31]. Considering these results, we designed and synthesized four bioconjugates, molecules associating pyrrole heterocycle and FELL peptide in order to search a synergic analgesic effect between both part of the molecule, taking into account the fact that pain is among the typical features of each inflammatory process, since many of the mediators of inflammation also mediate the pain [32]. Thus, the evaluation of analgesia could be regarded as an indirect criterion for the anti-inflammatory effect of the substance. In addition, this idea is supported by our previous studies on hybrid molecules containing pyrrole moiety coupled with peptides which revealed that newly created molecules have a similar or better activity compared to those of the parent compounds with multiple targeting [19]. 

## 2. Materials and Methods

### 2.1. Synthesis of Pyrrole Derivatives Containing COOH Group

The needed reagents for the synthesis of pyrrole derivatives, ethyl alcohol, chloroform, sulfuric acid, hydrochloric acid and sodium hydroxide, were purchased from Valerus (Sofia, Bulgaria). The starting substrate diethyl 2,4-dimethylpyrrole-3,5-dicarboxylate (CAS Number: 2436-79-5) is from Sigma-Aldrich (Ansbach, Germany). The synthetic reactions of the pyrrole compounds were controlled by Thin-Layer Chromatography (TLC) using aluminum plates covered with Silica gel 60 F_254_ (Merck, Darmstadt, Germany). All reagents were not additionally treated. 

#### 2.1.1. Synthesis of 4-(Ethoxycarbonyl)-3,5-dimethyl-1H-pyrrole-2-carboxylic Acid (A)

Alkaline hydrolysis according to the synthetic methodology described by Gossauer was used to synthesize targeted product A [33]. Briefly, 0.10 moles of diethyl 2,4-dimethylpyrrole-3,5-dicarboxylate were dissolved in 140 mL of ethanol and 70 mL of 25% NaOH was further added. The reaction was run in a boiling water bath and the development of the reaction was monitored by TLC (10:1/CHCl_3_:C_2_H_5_OH). Two hours later, the transformation was completed, the reaction mixture was cooled to around 22 °C and a further 20% HCl was added until pH 2.0 was achieved. The obtained crystalline residue was filtered off, washed with dH_2_O until pH 7.0 was achieved, dried under vacuum and recrystallized in warm ethanol. The obtained target product is a white solid with a yield 88%, m.p. 265–269 °C, and Rf 0.68 (10:1/CHCl_3_:C_2_H_5_OH).

#### 2.1.2. Synthesis of 5-(Ethoxycarbonyl)-2,4-dimethyl-1H-pyrrole-3-carboxylic Acid (B)

The targeted product B was synthesized by acid hydrolysis according to Gossauer’s methodology [33]. Briefly, 0.10 moles of diethyl 2,4-dimethylpyrrole-3,5-dicarboxylate were added in small portions while stirring to 80 mL conc. H_2_SO_4_, ensuring that the temperature does not exceed 40 °C. After adding the whole amount of the starting pyrrole, the mix was stirred for 20 more minutes at a temperature around 22 °C. Further, the reaction mixture was poured onto ice-distillated water. The crystalline residue was filtered off, washed with dH_2_O until pH 7.0 was achieved and suspended into 150 mL dH_2_O. NaOH (8%) was added to the suspension until a pH of around 8 was achieved. The insoluble part, which represents the unreacted starting product, was filtered off, and the filtrate was acidified with 30% H_2_SO_4_ till pH 3.0. The separated crystalline residue was filtered off, dried and recrystallized from ethanol. The obtained product is a brown solid with a yield 70%, m.p. 205–206 °C, and Rf 0.77 (10:1/CHCl_3_:C_2_H_5_OH).

### 2.2. Synthesis of Aimed Compounds Using Peptide Synthesis on Solid Support (SPPS)

Specifically protected amino acids with general formula ^α^N-Fmoc-Aaa(Xx)-COOH, where Aaa = L-Phe, L-Glu and L-Leu and Xx = *t*Bu (for Glu), Fmoc-Rink Amide MBHA and 2-Chlorotrityl chloride *(2-CTC)* resins as well as the activation agents HBTU, DIC and PyBOP were purchased from Iris Biotech (Wunsiedel, Germany). The same supplier delivered the necessary reagents trifluoroacetic acid (TFA), triisopropylsilane (TIS) and *N*,*N*′-diisopropylethylamine (DIPEA). The used solvents DMF and DCM were obtained from Valerus (Sofia, Bulgaria). 4-*N*,*N*-dimethylaminopyridine (DMAP) is from Sigma-Aldrich (Ansbach, Germany).

A 20 mL glass specifically constructed reactor for SPPS, purchased from Lipopharm.pl (Branch in Gdansk, Poland) is used for the manual synthesis of the targeted compounds. All calculations are carried out for 100 mg or 200 mg of final molecule and, in the reaction vessel, the needed quantity of Rink-amide MBHA (load 0.63 mmol/g, 200–400 mesh) or 2-CTC (load 1.55 mmol/g, 100–200 mesh) resin, respectively, is placed. Additionally, the first amino acid is bonded to the solid support by means of the methodology:

#### 2.2.1. Attachment of the First Amino Acid to the 2-CTC Resin

The 2-CTC resin was swelled into dry DCM (dried onto molecular sieve 4Å) for 30 min and the solvent was removed by filtration under vacuum. An amount of 1.2 eq. acid of the needed protected amino dissolved in dry DCM (10 mL per gram 2CTC-resin) and a few drops of DMF were added to the reactor. Further, 4 eq. of DIPEA was also added. Thus, the resulting mixture was stirred for 2 h. The solvents were removed by filtration and the resin with bonded first amino acid was washed with DMF (2 × 10 mL for 1 min) and DCM (2 × 10 mL for 1 min). After, the deprotection of the Fmoc group from the attached amino acid was performed via treatment with a solution of 20% piperidine in DMF for 20 min. After the reaction time, the solution was removed by filtration and the rest was washed with DMF (2 × 10 mL for 1 min) and DCM (2 × 10 mL for 1 min). Successful removal of the Fmoc group from the first amino acid (fully blue coloring of the resin aliquot in standard Kaiser test) shows the complete bonding of the first amino acid. In order to secure that all active groups of the resin are blocked, the resin is treated with a solution containing DCM:CH_3_OH:DIPEA in a ratio of 34:4:2 (*v*/*v*/*v*) to deactivate any unreacted chlorine atoms. Thus, further addition of the second amino acid could be implemented following standard SPPS condensation. 

#### 2.2.2. Attachment of the First Amino Acid to the Rink Amide MBHA Resin

The Rink Amide MBHA resin was swelled into dry DCM for 30 min and the solvent was filtered under vacuum. Then, a solution of 20% piperidine in DMF was introduced into the reactor to deprotect the Fmoc group from the resin. After 20 min of stirring, piperidine solution was removed by filtration under vacuum and the resin was washed with DMF (2 × 10 mL for 1 min) and DCM (2 × 10 mL for 1 min). The presence of free amino groups was proven using a positive Kaiser test result (blue color of resin beans). At the same time, C-terminal amino acid is separately activated in a Becher glass, where 3 eq. of the amino acid were dissolved in 10 mL mixture of DMF/DCM (7:3). Further, 9 eq. of DIPEA and 3 eq. of HBTU were introduced. The obtained solution is mixed for 20 min and thrown in the deprotected resin. The reaction mix is stirred for another 4 to 6 h. Successful coupling of the amino acid was monitored using the Kaiser test (uncolored resin beans).

### 2.3. Synthesis of Targeted Bioconjugates

In a final step after the deprotection of the Fmoc group from the fully synthesized tetrapeptide on the solid support using 20% pyridine/DMF, the pyrrole derivative possessing free COOH was added using the standard coupling procedure. 

### 2.4. Final Deprotection and Releasing from the Resin

The final releasing from the resin is performed by two different treatments depending on the type of used resin:

- For 4 h with a mixture of 95% TFA, 2.5% TIS, and 2.5% dH_2_O when the Rink Amide MBHA resin is used;
- For 4 h with a mixture of 50% TFA/50% dH_2_O:TIS (97.5:2.5 eq.) for 2-CTC resin.

In both cases, at the end of the reaction time, resin is removed by filtering and the solvents were removed in vacuum and co-evaporate several times with n-hexane:DCM (1:1). The raw products are oils and they are further precipitated in cold, dry diethyl ether. 

### 2.5. Analysis of Targeted Molecules

HPLC was used to monitor the purity of all newly synthesized target molecules. Mass spectrometry on Shimadzu LC-MS/MS 8045 system (Shimadzu Corporation, Kyoto, Japan) was used to prove the structures of all molecules. In addition, the chromatographic system was equipped with an Agilent Poroshell 120 (Santa Clara, CA, USA), where the 100 mm × 4.6 mm column is worked at 40 °C. Mobile phase A: H_2_O (10% AcCN; 0.1% HCOOH) and B: AcCN (5% H_2_O, 0.1% HCOOH) with 0.30 mL/min was used. The gradient of the phase was 80%A/20%B, passes through 5%A/95%B in 15 min and returns to 80%A/20%B in 22 min. 

The MS was used in SCAN /ESI+ mode ionization with 3 L/min of the nebulizing gas flow, 10 L/min of the heating and drying gas flow, 350 °C interface temperature, 200 °C DL temperature and 400 °C heat block temperature. 

An automatic standard polarimeter Polamat A, Carl Zeis, Jena (Anton Paar Opto Tec GmbH, Seelze, Germany), was used to measure the optical rotation constant in dimethyl sulfoxide at c = 1. 

A semi-automatic melting point meter M3000 by A. KRÜSS Optronic GmbH (Hamburg, Germany) was used to determine melting temperatures. All constants are presented in Table 1 without any corrections.

IR analysis of both obtained pyrrole acids A and B were registered on a Varian 660 IR FT-IR Spectrometer(Agilent Technologies, Santa. Clara, CA, USA) in a KBr tablet and the obatained spectra are presented in the Figures S1 and S2. 

### 2.6. Hydrolytic Stability Study

In order to study the hydrolytic stability of the newly synthesized compounds, three model pH systems that mimic conditions in the stomach, blood plasma and small intestine were created. Model solutions including specific enzymes used for the determination of hydrolytic stability were prepared according to the *European Pharmacopoeia*, 6th Edition as follows:

i. pH 2.0 model system—Solution A: An amount of 6.57 g KCl in a CO_2_ free water and 119.0 mL 0.1 mol/L HCl are mixed in the volumetric flask and the final solution is completed to 1000.0 mL with dH_2_O. Solution B: An amount of 5 mg of pepsin was added to a 10 mL volumetric flask and completed to the mark with solution A. Thus, the final model solution is pH 2.0 and has a 0.5 mg/mL concentration of the trypsin.
ii. pH 7.4 model system—Amounts of 0.1 g of trypsin, 2.38 g Na_2_HPO_4_, 0.19 g KH_2_PO_4_ and 8.0 g NaCl are dissolved to a total of 1000.0 mL in dH_2_O in order to obtain a 0.1 mg/mL final concentration of trypsin in a model system with pH 7.4. An amount of 1 ml blood plasma (ACCUCLO^TM^ Reference plasma, Normal, Sigma Diagnostics) is the recovery with 15 mL of obtained buffer with pH 7.4.
iii. The pH 9.0 model system is obtained by mixing of 1000 mL of the solution containing 6.18 g H_3_BO_3_ in 0.1 mol/L KCl in dH_2_O and 420.0 mL of solution containing 0.1 mol/L NaOH in dH_2_O. A further 0.1 mg of trypsin is dissolved in the solution with pH 9.0 and completed to 10 mL in a volumetric flask in order to obtain the final concentration of 0.1 mg/mL of the trypsin.

The hydrolytic stability is determined using a Perkin-Elmer series 200 HPLC (Waltham, MA, USA), Lichrospher RP-8 Non Endcpd column with pore size 5 µm, i.d. 4.6 mm and 150 mm length (Alltech, Lexington, KY, USA), UV detection at 274 nm (PerkinElmer series 200 detector, Waltham, MA, USA), room temperature, injection volume of 20 μL and flow rate of 0.70 mL/min, with the gradient at 0.0 min at 20% B, at 10 min at 100% B, at 10 to 13 min at 100% B, at 13 to 14 min at 20% B and at 16.5 min at 20% B. The Mobile phases were as follows: Solution A: Acetonitrile/Water/TFA—5:95:0.1; Solution B: Acetonitrile/Water/TFA—95:5:0.1.

### 2.7. In Vivo Analysis 

#### 2.7.1. Experimental Animals

The male Wistar rats (180–200 g) were used for all experiments. All animals were kept at around 22 °C and under normal conditions. The experimental procedures are realized in the interval 10:00 a.m. to 1:00 p.m. and are approved by the Research Ethics Commission of the Medical University-Sofia.

#### 2.7.2. Analgesic Activity Evaluation

Several groups of experimental animals were used. Each group contains 8–10 rats:

- The control group receives 0.2 mL of saline intraperitoneally injected (i.p.);
- Six experimental groups receiving one of the 6 tested substances (0.25 mg/kg, i.p.)—pyrrole acid A, pyrrole acid B, BB19A, BB19B, BB20A, and BB20B;
- Four experimental groups receiving Naloxone (1 mg/kg, i.p.) before the administration of the newly synthesized bioconjugates BB19A, BB19B, BB20A, and BB20B;
- Four experimental groups receiving AM251 (1.25 mg/kg, i.p.) before the administration of the newly synthesized bioconjugates BB19A, BB19B, BB20A, and BB20B.

Pretreatments with the non-selective opioid receptor antagonist Naloxone or the cannabinoid receptor antagonist AM251 were performed in order to estimate the types of receptors engaged in the effects observed—respectively, the opioid [34] or the cannabinoid receptor [35].

#### 2.7.3. Nociceptive Paw Pressure Test (Randall–Selitto Test)

An analgesiometer was used to measure the specific changes in the mechanical nociceptive thresholds (PPT) of experimental animals [36]). A pressure was applied to the hind-paw and the value (g) required to elicit a nociceptive response (such as squeak or struggle) was taken as the mechanical nociceptive threshold. A cut-off value of 500 g was used to prevent damage of the paw. The targeted compounds were administrated and further nociception was evaluated each 10 min from the 10th min until the 50th min.

#### 2.7.4. Data Analysis

The results were statistically assessed by one-way analysis of variance followed by Newman-Keuls post hoc comparison test. One-Way ANOVA has been used to analyze the data. Values are represented in arbitrary units (AU) according to the scale, as mean ± S.E.M. Values of *p* < 0.05 were considered to indicate statistical significance. 

## 3. Results

### 3.1. Synthesis of Pyrrole Acids **A** and **B**

**Figure 2:**
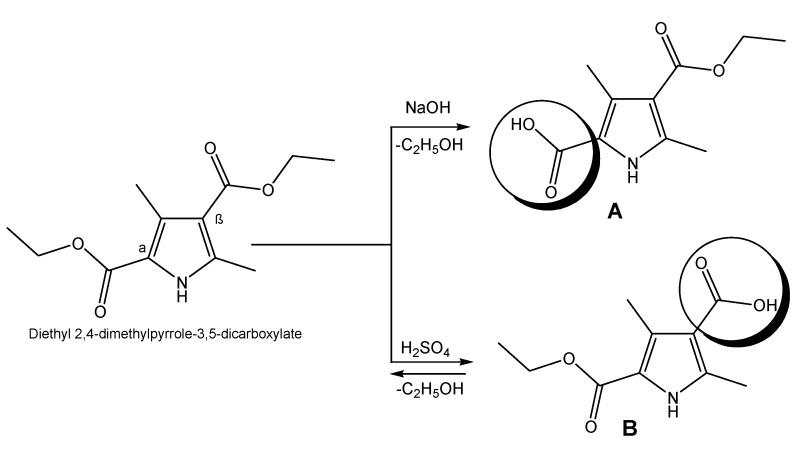
Synthesis of pyrrole derivatives possessing COOH group.

Targeted pyrroles are synthesized using a standard procedure of saponification in order to obtain the necessary derivatives with the free COOH group, which will be further used for coupling to the peptide moiety. Hydrolysis of esters occurs in both acidic and alkaline media depending on the pyrrole acid structure [37]. In the presence of a hydroxide (NaOH), the yield of compound **A** is close to 100%, whereas in acidic medium (H_2_SO_4_), the reaction is in equilibrium and the conversion of obtaining **B** is incomplete (Figure 2).

### 3.2. Synthesis and Characterization of Targeted Bioconjugates

**Figure 3:**
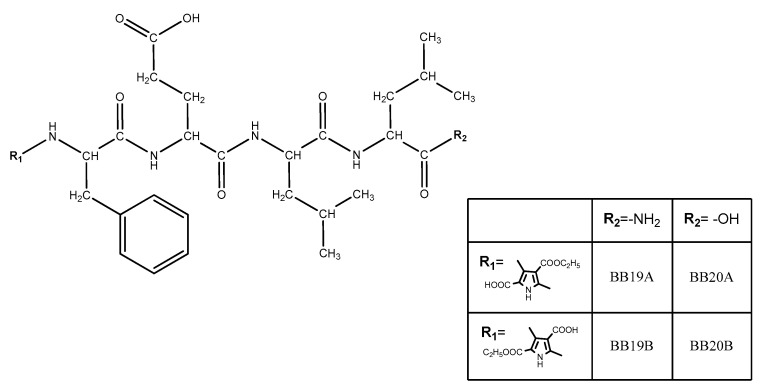
General structure of newly synthesized bioconjugates.

Four new peptide-based bioconjugates were synthesized with the general formula presented in Figure 3. Previously in vivo-evaluated tetrapeptides FELL-OH and FELL-NH_2_ and pyrrole moiety (R_1_ = **A** or **B**) were combined in the structures of targeted hybrid entities in order further to study their potential analgesic activity and to evaluate the role of both combined entities for the activity.

Four newly synthesized compounds are fully characterized and the data are summarized in Table 1.

**Figure 4:**
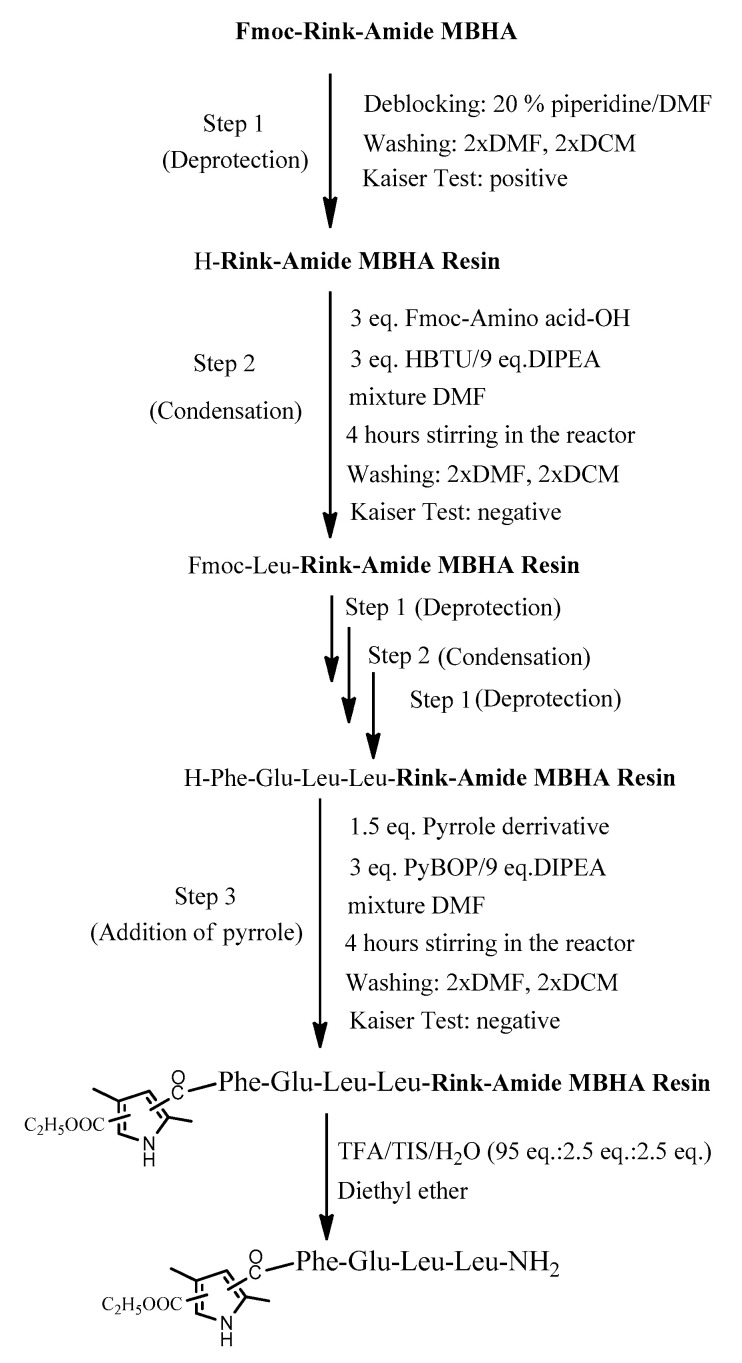
General synthesis of **BB19A-B** using Rink-Amide MBHA Resin.

**Figure 5:**
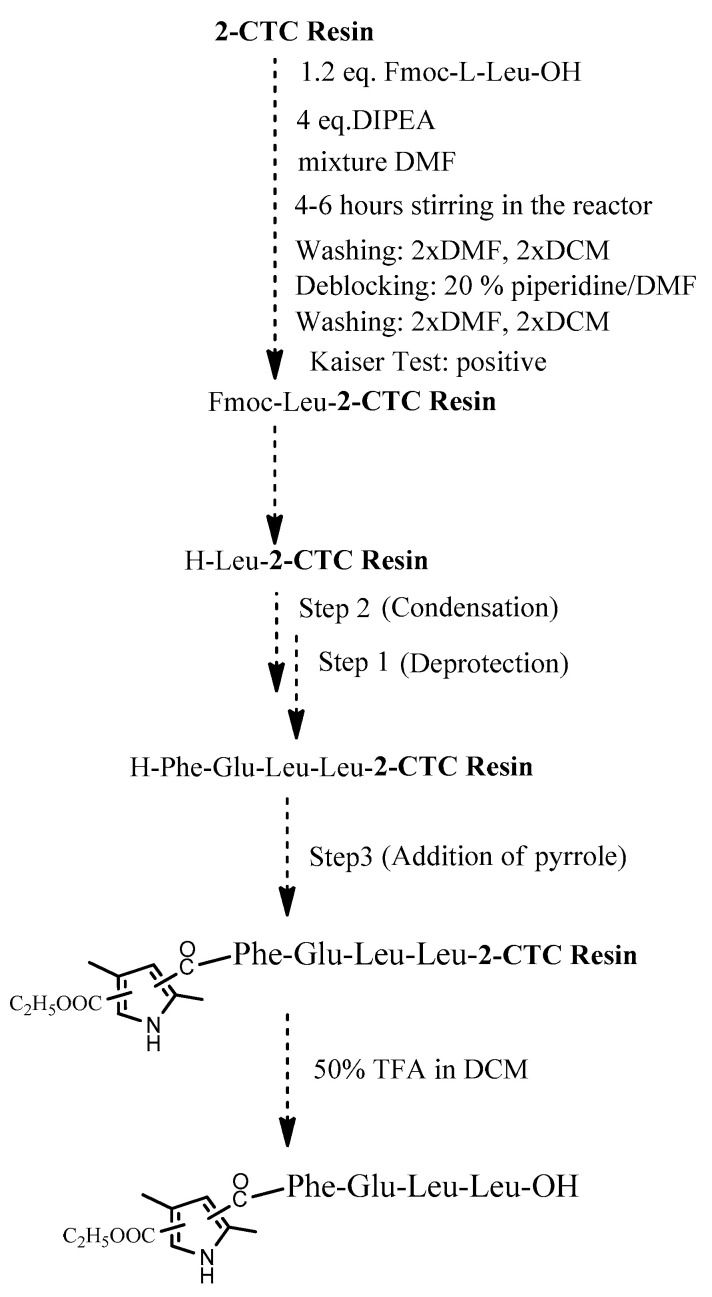
General synthesis of **BB20A-B** using 2-CTC Resin.

The four target compounds were synthesized according to general schemes presented in Figure 4 for **BB19A-B** and Figure 5 for **BB20A-B**.

Using 2-CTC resin for the synthesis of C-terminal free acids has numerous advantages. Especially, it effectively prevents racemization when incorporating the first protected amino acid and prevents the reaction of peptide cleavage from the resin in some specific amino acid sequences [38]. In addition, it is more activated for the first condensation reaction rather than standard CTC-resin. Thus, this resin has gained widespread popularity in SPPS. 

### 3.3. Study of the Analgesic Activity of the New Compounds

**Figure 6:**
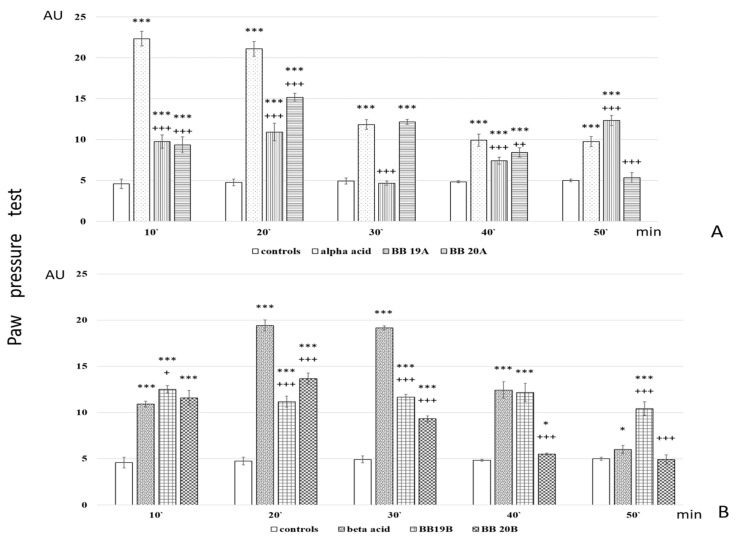
Analgesic activity of (**A**) Pyrrole acid **A** and its derivatives **BB19A** and **BB20A** and (**B**) Pyrrole acid **B** and its derivatives **BB19B** and **BB20B**, estimated by Paw pressure test. Paw pressure thresholds are presented in arbitrary units (AU) as mean values ± S.E.M. *** *p* < 0.001, * *p* < 0.05 vs. controls; ^+++^ *p* < 0.001, ^++^ *p* < 0.01, ^+^ *p* < 0.05 vs. acid **A** (**A**) or acid **B** (**B**), respectively.

Analgesic activity of targeted bioconjugates was monitored by using the Paw pressure test (Randall–Selitto test) presented in the Materials and Methods section. The obtained results of both pyrrole free acids **A** and **B** and peptide-based bioconjugates containing pyrrole moiety are presented in Figure 6.

**Figure 7:**
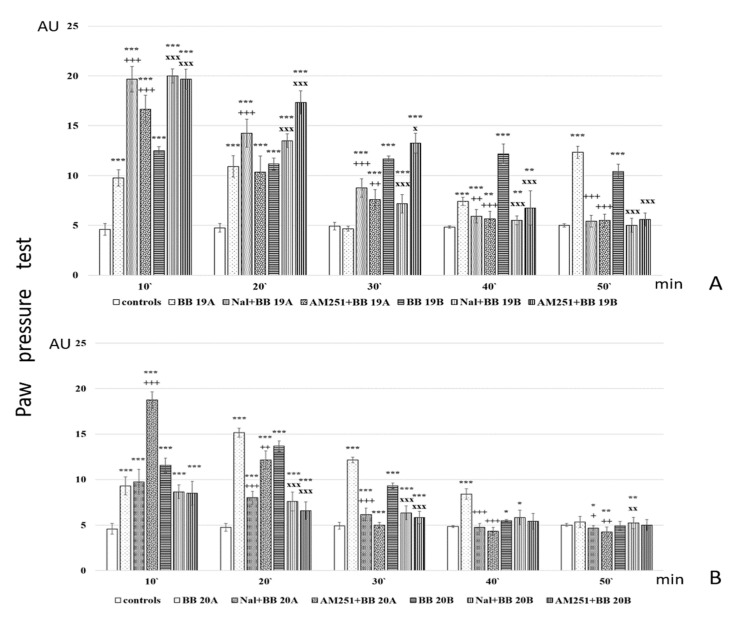
Analgesic activity of (**A**) **BB19A** and **BB19B**, and (**B**) **BB20A** and **BB20B** after Naloxone or AM251 pretreatment, estimated by Paw pressure test. Paw pressure thresholds are presented in arbitrary units (AU) as mean values ± S.E.M. *** *p* < 0.001, ** *p* < 0.01, * *p* < 0.05 vs. controls; Naloxone and AM251 pretreatments have been compared with the bioconjugate without pretreatment: ^+++^ *p* < 0.001, ^++^ *p* < 0.01, ^+^ *p* < 0.05 vs. **BB19A** (**A**) or **BB20A** (**B**), respectively; ^xxx^ *p* < 0.001, ^xx^ *p* < 0.01, ^x^ p < 0.05 vs. **BB19B** (**A**) or **BB20B** (**B**), respectively.

Further, analgesic activity after pretreatment with AM251 and Naloxone was also studied. The obtained results are presented in Figure 7.

### 3.4. Hydrolytic Stability Study

**Figure 8:**
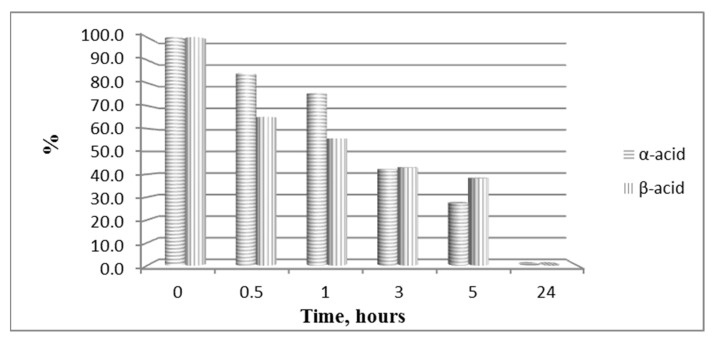
Hydrolysis of both pyrrole acids at pH 2.0.

**Figure 9:**
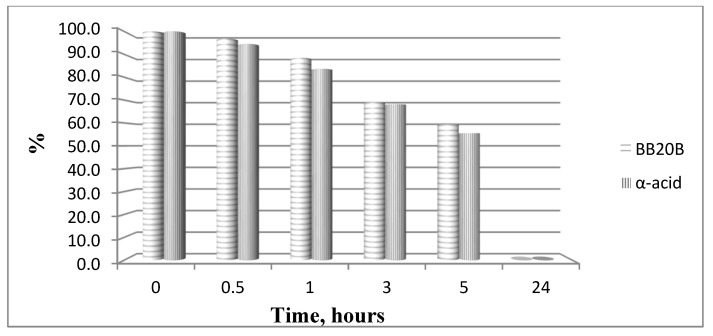
Hydrolysis of targeted compounds **BB20B** and α-pyrrole acid at pH 7.4.

The hydrolytic stability of the obtained bioconjugates was evaluated in specifically designed model systems which mimic three parts of the human organism stomach (pH 2.0), blood plasma (pH 7.4) and small intestine (pH 9.0), and the results for all hydrolytically unstable compounds in model systems are presented in Figure 8 and Figure 9. 

## 4. Discussion

There are many literature data that suggest pyrrole heterocyclic derivatives exhibit a wide range of biological effects such as antidepressant, antipsychotic, antituberculosis, antihypertensive, anticoagulant, antiviral, antimicrobial, anticonvulsant, anticancer and analgesic effects [39,40,41,42]. In addition, this heterocyclic ring participates in the pharmacophore system of a large number of active compounds previously synthesized by our group [19,43]. Thus, herein, bioconjugates including pyrrole moieties were designed, synthesized and studied for their analgesic activity using the scheme presented in Figure 2. An alternative dependence on the pH of the medium was observed, where the alkaline medium vectorizes the hydrolysis of the α-standing ester group, and the acidic medium favors the hydrolysis of the β-standing ester group. Probably, the hydrolysis of α-esters in an alkaline environment could be explained by their stabilization by means of an intramolecular bridge. The obtaining of the free COOH function was proven using the FT-IR of the starting ester and final free acids. The obtained data from the analysis show the presence of a large stretch band between 3200 and 2500 cm^−1^ which was missed in the FT-IR spectrum of the starting diester (Figures S1 and S2). 

**Figure 10:**
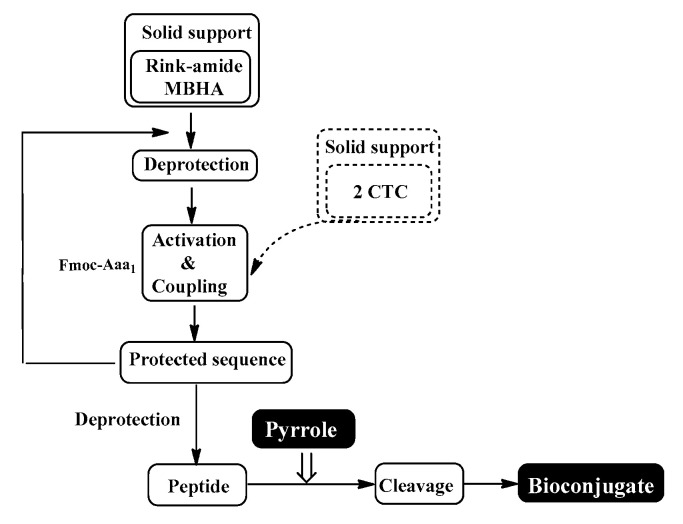
Schematic representation of conventional SPPS cycle.

The conventional manual SPPS was used for the synthesis of targeted bioconjugates. Thus, protected amino acids are consecutively added to a growing peptide chain immobilized on a solid support (Rink-amide MBHA or 2 CTC resin) from the C- to N-terminus (Figure 10), according to the methodologies described in the Material and Methods section.

The aimed peptide sequences are obtained by repeating deprotection and condensation steps using the sequential addition of the appropriate protected amino acids, immobilized on one of the corresponding suitable resins for C-terminal derivative synthesis. Each step is monitored using a standard Kaiser test (briefly, blue resin beans for a positive result for deprotection and uncolored resin beans for a successful condensation). Once the synthesis of FELL-peptide is completed, pyrrole heterocycle was added to obtain the desired conjugates. For the synthesis of targeted peptides and bioconjugates, Rink-amide MBHA or 2-CTC resins depending on the C-terminal modification were used as the solid-phase carrier. HBTU or DIC were used for the condensation step and, respectively, DIPEA or DMAP as catalysts. PyBOP was applied to activate the pyrrole scaffolds **A** and **B** at the final stage of synthesis of the targeted bioconjugates. The coupling reactions were performed using amino acid/HBTU(PyBOP)/DIPEA/resin in a molar ratio 3/3/9/1 or amino acid/DIC/resin in a molar ratio 3/3/1 and catalytic quantity of DMAP. The N^α^-Fmoc-group was deprotected on every stage by treatment with 20% piperidine solution in DMF. The cleavage of aimed bioconjugate–amides from the Rink Amide MBHA resin was performed with a mixture of 95% TFA, 2.5% TIS and 2.5% dH_2_O. The cleavage from the 2-CTC resin for C-terminal free acid synthesis was conducted using a mixture of 50% TFA and 50% dH_2_O. The synthesized bioconjugates were obtained as oils further precipitated through the addition of cold dry diethyl ether. The *C*-terminal amides were obtained in a good yield of more than 89%, whereas *C*-terminal free acids were synthesized with moderate yields between 27 and 49%.

Parent compound FELL (Phe-Glu-Leu-Leu-OH) and four analogs of the tetrapeptide were previously evaluated in vivo for their analgesic activity [44]. The performed experiments showed that Leu is the best choice as hydrophobic amino acid in positions 3 and 4 of the sequence. In addition, the modification of C-terminal carboxyl function to amide one (Phe-Glu-Leu-Leu-NH_2_) gradually increased analgesic activity with time. However, tetrapeptide with the C-terminal free acid group demonstrated a constantly increasing analgesic activity for the total time of the evaluation.

Analysis of in vivo studies reveals that the newly synthesized hybrid entities **BB19A**, **BB20A**, **BB19B**, and **BB20B** exhibited analgesic activity, which was, interestingly, rather stable throughout the experience although generally lower than that of the starting substances alpha- and beta-pyrrole acids, respectively, for short action times (Figure 6A,B). **BB19A** exhibited biphasic activity that, on the 50th min, exceeded the parent alpha acid (F = 38.83498) (Figure 6A), while **BB19B** exhibited sustained analgesic activity that, on the 10th (F = 4.67914) and 50th min (F = 296.52941), was also more significant than the parent beta acid (Figure 6B). The FELL-OH-peptide containing **BB20A** and **BB20B** bioconjugates also expressed analgesic activity. For **BB20A**, PPTs were highest on the 20th min; after that, they decreased to control values (Figure 6A). PPTs after **BB20B** on the 10th min were equivalent to the parent beta acid’s ones, but did not show a substantial increase on the 20th min, and slowly decreased to control levels (Figure 6B). After the opioid receptor blockade, the analgesic activity of the FELL-OH-peptide-containing bioconjugates decreased, while **BB19A** and **BB19B** led to significant analgesia that even initially exceeded that of the substances without the pretreatment (Figure 7A,B). Pretreatment with AM251 somehow showed similar results. A paradoxically higher (than without pretreatment) analgesia (as the one described after naloxone) resulted on the 10th min for **BB19A**; on the 10th, 20th, and 30th min for **BB19B** (Figure 7A); and also for **BB20A** on the 10th min (Figure 7B). 

In order to study the hydrolytic stability of newly synthesized molecules, all hybrid structures were dissolved in buffer with a final concentration of 1 mg/mL. After shaking for 10 min, internal standard at a concentration of 1 mg/mL was added. In the analysis in the model system with pH 2.0 pepsin (dissolved in buffer pH 2 with a concentration 0.5 mg/mL) was added to give a pepsin/peptide ratio of 1:20. In the model system with pH 7.4, a recovery blood plasma was used to obtain final concentration of peptide, 1 mg/mL. In the model system with pH 9, trypsin (dissolved in buffer pH 9.0, with a concentration of 0.1 mg/mL) was added to give an enzyme/peptide ratio of 1:100. Samples were placed in a shaking incubator (500 rpm, 37 °C) and aliquots of 20 µL were analyzed after 0, 0.5, 1, 3, 5, and 24 h. All peptides were completely stable except for acids **A** and **B** in pH 2.0 and acid **B** and peptide **B20B** in pH 7.4, as it is shown in Figure 8 and Figure 9.

The following structure–activity relationships can be established:

- Both acids **A** and **B** show short-lasting analgesic activity (of about 10 min)—between the 10th and 20th min for acid **A**, and between the 20th and 30th min for acid **B**;
- Acid A retains its analgesic activity (even with a 56% decrease) until the end of the estimated time, while on the 50th min, PPTs for acid **B** are almost equivalent to the controls’;
- All four of the newly synthesized bioconjugates possess analgesic activity, which results in being inferior (with a few exceptions) to that of the parent acids **A** and **B**;
- The pyrrole A-containing bioconjugate **BB20A** reaches the highest analgesic activity, with more than 87% of that of the parent acid **A**;
- An interesting biphasic analgesic activity has been observed for of **BB19A** that appears to be naloxone-independent in the first phase and naloxone-dependent in the second;
- **BB19B** presents with the most constant analgesic activity—varying less than 7% during the whole time of the experiment, and reaching 64.4% of the parent acid **B** analgesic activity;
- Opioid receptors appear to be involved in the analgesic effects of the FELL-OH-peptide-containing (**BB20A** and **BB20B**) bioconjugates;
- Cannabinoid receptors are also involved in the analgesic effects of the FELL-OH-peptide-containing (**BB20A** and **BB20B**) bioconjugates, but with **BB20A,** they “turn on” at a later stage—after the 20th minute;
- Neither naloxone nor AM251 succeeded in preventing some analgesic activity after the pretreatments—the two types of receptors, opioid and cannabinoid, obviously are synergistic in their analgesic effects, and even some other types of receptors, e. g. NOP, could be involved;
- A paradoxical analgesic activity (higher than the one without naloxone pretreatment) has been observed for the compounds **BB19A** and **BB19B** that could be due to the anti-opioid effects of the compounds, as well as the involvement of another type of receptor, e.g., NOP, in the described effects.

All bioconjugates are stable in the used model systems for hydrolytic stability investigation except acids **A** and **B** in pH 2.0 and acid **B** and peptide **BB20B** in pH 7.4.

## 5. Conclusions

In a short conclusion, the obtained results reveal that all newly synthesized bioconjugates have analgesic activity according to the used Paw pressure test. Although free pyrrole acids showed the best analgesic activity, they are the most unstable to the hydrolysis. Combination with peptide structure leads to the hydrolytic stabilization of the bioconjugates, albeit with slightly reduced activity. 

## Figures and Tables

**Table 1:** Characteristic data for the new bioconjugates.

Code	Structure	Molecular formula	Mm _exact,_ g/mol	[M+nH]^+^_observed_	[M+Na]^+^_observed_	M.p., °C	t_R_, min	α_D_^20^, ^O^	Yield, %	Chromatographic purity, %
**BB19A**	A-Phe-Glu-Leu-Leu-NH_2_	C_36_H_52_N_6_O_9_	712.38	713.35	735.35	213±215	5.600	13	89	85
**BB19B**	B-Phe-Glu-Leu-Leu-NH_2_	C_36_H_52_N_6_O_9_	712.38	713.35	733.35	207±208	6.567	8	89	100
**BB20A**	A-Phe-Glu-Leu-Leu-OH	C_36_H_51_N_5_O_10_	713.36	714.35	736.30	231±233	7.350	-21	49	88
**BB20B**	B-Phe-Glu-Leu-Leu-OH	C_36_H_51_N_5_O_10_	713.36	714.35	736.30	214±216	6.833	34	27	100

## Data Availability

Some amounts of the molecules are available from the corresponding author.

## Supplementary Materials

The following supporting information can be downloaded at: https://www.mdpi.com/article/10.3390/biomedicines11123265/s1, Figure S1: FT-IR spectra of: 0-2,4-dimethylpyrrole-3,5-dicarboxylate; 1-4-(Ethoxycarbonyl)-3,5-dimethyl-1H-pyrrole-2-carboxylic Acid (A); Figure S2: FT-IR spectrum of 5-(Ethoxycarbonyl)-2,4-dimethyl-1H-pyrrole-3-carboxylic Acid (B).
