# Supplementary figures and images for: Synthesis, Hydrolytic Stability and In Vivo Biological Study of Bioconjugates of the Tetrapeptides FELL Containing Pyrrole Moiety
Source: Biomedicines. 2023 Dec 9;11(12):3265. doi: 10.3390/biomedicines11123265 (PMC10740831; doi:10.3390/biomedicines11123265)

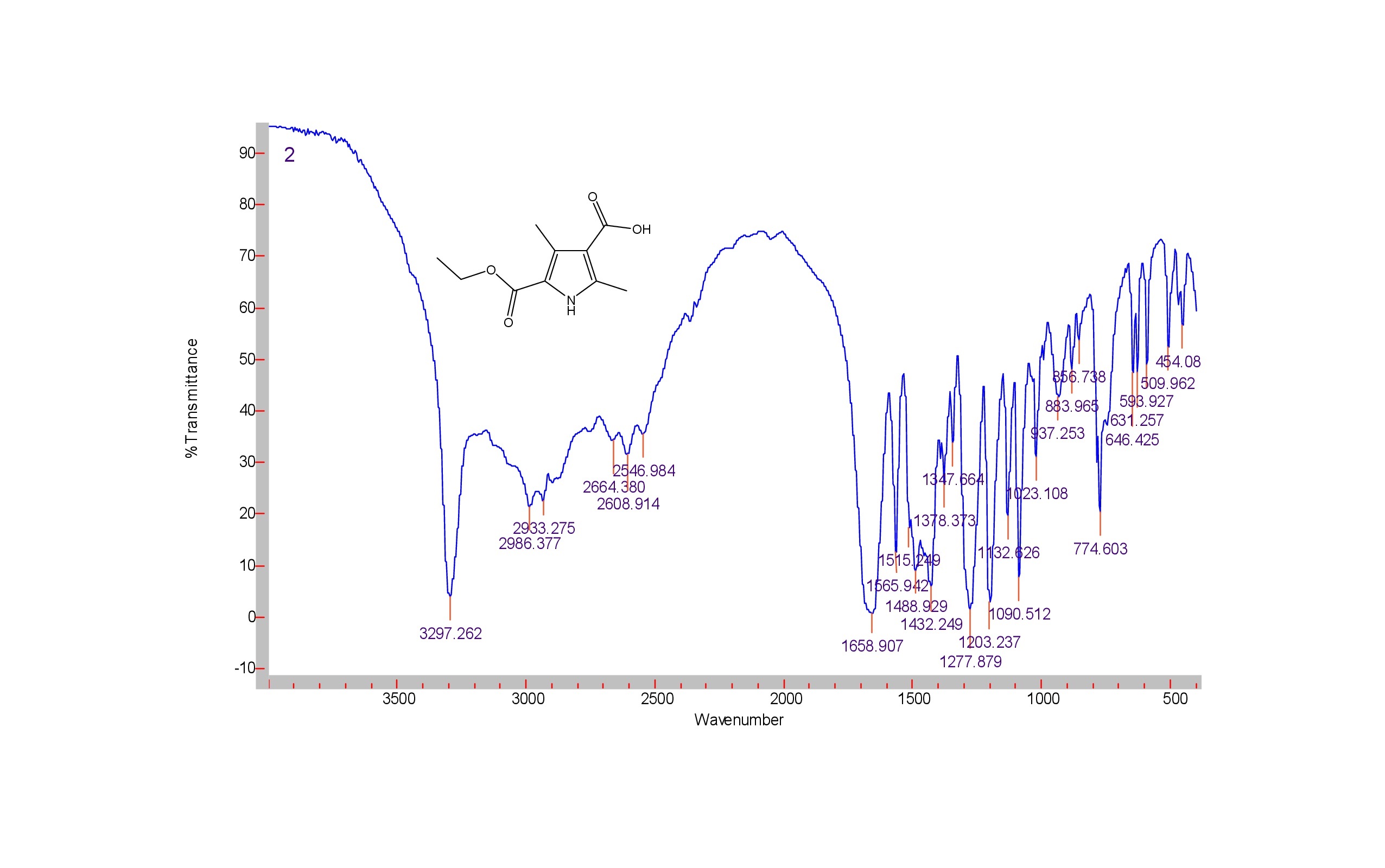

Supplement: Supplementary file 1 [file biomedicines-11-03265-s001.zip › FT-IR supplamentary files-0002.jpg]

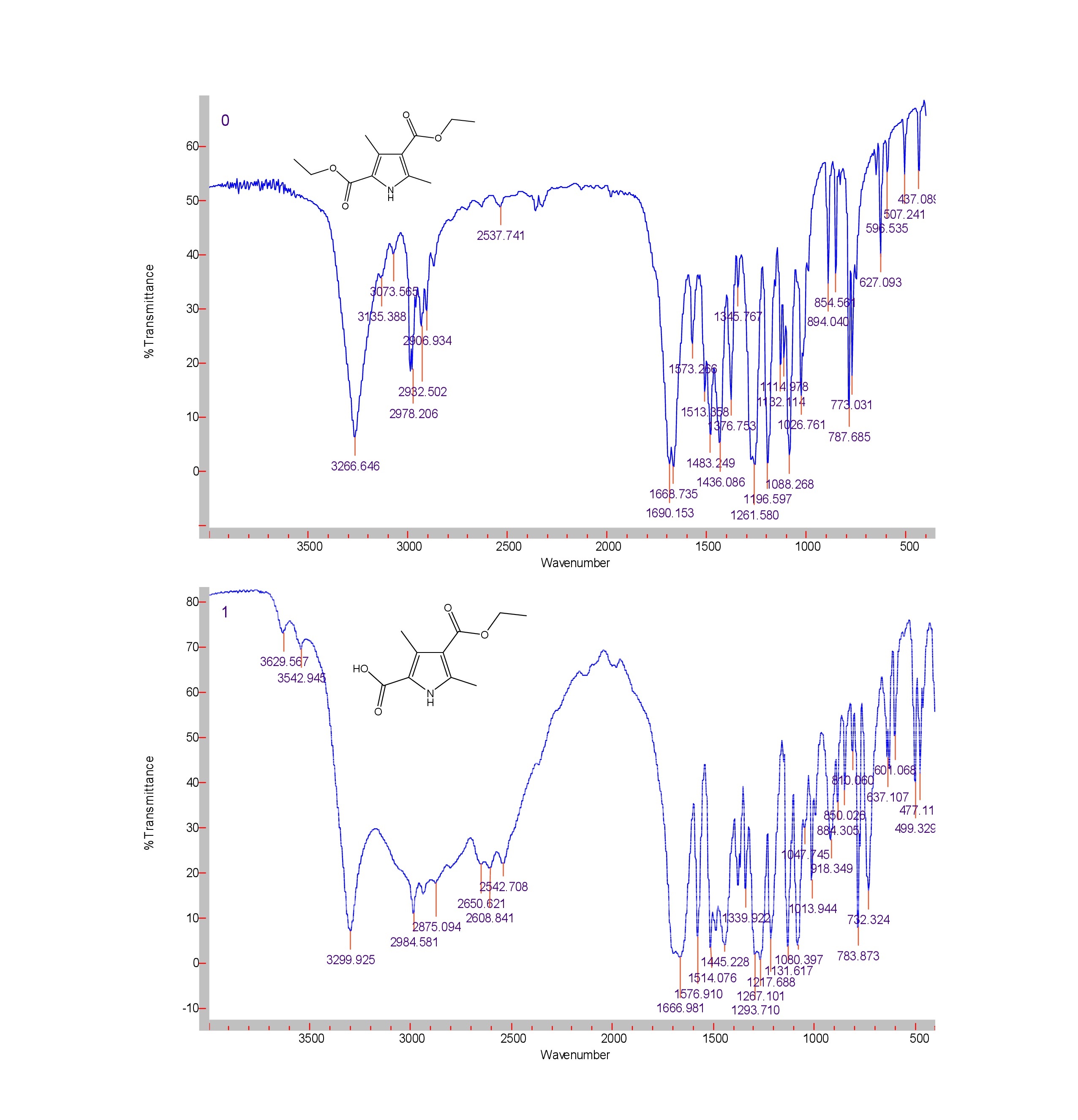

Supplement: Supplementary file 1 [file biomedicines-11-03265-s001.zip › FT-IR supplamentary files-0001.jpg]
